# Probing remote residues important for catalysis in *Escherichia coli* ornithine transcarbamoylase

**DOI:** 10.1371/journal.pone.0228487

**Published:** 2020-02-06

**Authors:** Lisa Ngu, Jenifer N. Winters, Kien Nguyen, Kevin E. Ramos, Nicholas A. DeLateur, Lee Makowski, Paul C. Whitford, Mary Jo Ondrechen, Penny J. Beuning

**Affiliations:** 1 Department of Chemistry & Chemical Biology, Northeastern University, Boston, MA, United States of America; 2 Department of Physics, Northeastern University, Boston, MA, United States of America; 3 Department of Bioengineering, Northeastern University, Boston, MA, United States of America; University of Connecticut, UNITED STATES

## Abstract

Understanding how enzymes achieve their tremendous catalytic power is a major question in biochemistry. Greater understanding is also needed for enzyme engineering applications. In many cases, enzyme efficiency and specificity depend on residues not in direct contact with the substrate, termed remote residues. This work focuses on *Escherichia coli* ornithine transcarbamoylase (OTC), which plays a central role in amino acid metabolism. OTC has been reported to undergo an induced-fit conformational change upon binding its first substrate, carbamoyl phosphate (CP), and several residues important for activity have been identified. Using computational methods based on the computed chemical properties from theoretical titration curves, sequence-based scores derived from evolutionary history, and protein surface topology, residues important for catalytic activity were predicted. The roles of these residues in OTC activity were tested by constructing mutations at predicted positions, followed by steady-state kinetics assays and substrate binding studies with the variants. First-layer mutations R57A and D231A, second-layer mutation H272L, and third-layer mutation E299Q, result in 57- to 450-fold reductions in *k*_cat_/*K*_M_ with respect to CP and 44- to 580-fold reductions with respect to ornithine. Second-layer mutations D140N and Y160S also reduce activity with respect to ornithine. Most variants had decreased stability relative to wild-type OTC, with variants H272L, H272N, and E299Q having the greatest decreases. Variants H272L, E299Q, and R57A also show compromised CP binding. In addition to direct effects on catalytic activity, effects on overall protein stability and substrate binding were observed that reveal the intricacies of how these residues contribute to catalysis.

## Introduction

**Fig 1:**
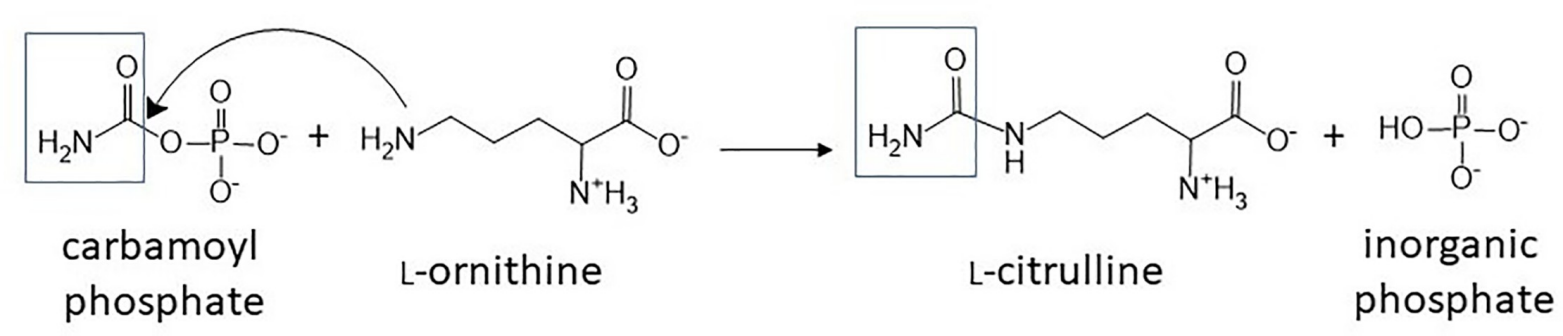
OTC catalysis. OTC catalyzes the reaction of carbamoyl phosphate and ornithine to generate citrulline and inorganic phosphate. In the presence of iron (III) chloride and TSC, under acidic conditions, 2,3-butadione monoxime reacts with citrulline that results in formation of a pink color detected at 530 nm [12–14].

Ornithine transcarbamoylase (OTC) is an essential enzyme in the urea cycle and in the arginine biosynthesis pathway that catalyzes the formation of citrulline (CIT) and inorganic phosphate (P_i_) from carbamoyl phosphate (CP) and L-ornithine (ORN) (Fig 1) [1–3]. Anabolic OTC encoded by the *E*. *coli argI* gene functions as a homotrimer of 333-residue subunits [4, 5]. Each subunit possesses a CP binding domain consisting of N-terminal residues 1–132 and an ORN binding domain consisting of C-terminal residues 149–310 that are connected through helices H5 (residues 133–148) and H13 (residues 311–333) [3, 6, 7]. OTC utilizes an ordered bi-bi substrate mechanism with CP binding first, followed by ORN and the sequential release of CIT and then P_i_. [3] During catalysis, OTC is reported to undergo an induced-fit conformational change upon binding CP that significantly contributes to its rate [3, 8]. Evidence cited for this conformational change includes results from OTC crystal soaking with CP and the bisubstrate analog N-phosphonoacetyl-L-ornithine (PALO), and fluorescence and UV difference binding assays [1, 2, 9]. The CP-induced conformational change of human OTC (huOTC) also contributes to the sequential binding of the zwitterion form of ORN, with a positively charged α- or δ-amino group, for optimal catalysis [10, 11].

HuOTC shares ~49% sequence homology with *E*. *coli* OTC and both function as homotrimers with masses of ~110 kDa [2, 7]. As of 2015, 417 mutations in huOTC were characterized to be associated with OTC deficiency (OTCD) [15], resulting in buildup of ammonium, glutamine, additional amino acids, and orotic acid [16, 17]. OTCD is the most common urea cycle disorder, estimated to occur in ~1 of 14,000 to ~1 of 77,000 newborns [18, 19]. OTCD patients experience personality dysfunction, intellectual impairment, cerebellar ataxia, encephalitis, seizures, coma and death [16, 17, 20]. Mutations of the OTC gene lead to impaired or inactive OTC catalysis that causes a broad spectrum of OTCD symptoms [20–22].

Active site residues are often inferred from x-ray crystal structures, which have been critically important in the elucidation of catalytic mechanisms of enzymes. In the identification of functionally active amino acid residues, both mutagenesis experiments and inferences from structural studies have focused on the residues that interact directly with the substrate molecule(s). Recently, computational predictions have suggested that enzyme active sites can be spatially extended, with amino acids in the second and third layers around the bound substrate molecule(s) contributing significantly to catalysis [23–27]. The example reported herein adds to the body of recent experimental evidence [23–27] that distal residues participate in catalysis and that the specific residues that participate can be identified computationally. Establishing the involvement of these distal residues, and understanding how they influence catalysis, are important for elucidating how natural enzymes work and for the development of design principles for protein engineering.

Partial Order Optimum Likelihood (POOL), a machine learning methodology we have developed, utilizes the three-dimensional structure of a protein to predict catalytically important residues based on computed, residue-specific electrostatic and chemical properties [23, 28–31]. POOL is a multidimensional, isotonic regression with a monotonicity constraint, i.e. the outcome (in this case the probability that a specific amino acid participates in the biochemical activity) is a monotonic function of the input features. Input parameters [29] that can be used with POOL to predict functionally important residues include: (1) Residue-specific chemical information from THEMATICS, which predicts functionally important residues based on electrostatic properties [32–36]; (2) Phylogenetic information from INTREPID, which predicts functionally important residues based on the phylogenetic tree [37]; and (3) Surface topology information from the structure-only version of ConCavity, which predicts catalytic sites based on surface topological properties of a protein [38]. POOL generates a rank-ordered list of all the amino acids in the protein structure, according to their likelihood of involvement in catalysis and/or ligand binding. POOL has been shown to: (1) accurately predict catalytic residues of an enzyme and (2) discern between spatially compact and extended active sites that include remote residues [23, 24, 26, 31]. Here, POOL is used as a guide to identify catalytically active residues in *E*. *coli* OTC and these residues are studied with site-directed mutagenesis and kinetics and binding assays to understand the role these residues play in catalysis. Conservative mutations were made to selected residues, including POOL-predicted and control residues, and the resulting variants were characterized for their catalytic efficiency, CP binding ability, and overall protein stability, conformation, and dynamics. We find five POOL-predicted residues located outside the first layer that do not interact directly with the substrate molecules, but contribute substantially to efficient catalysis. We define second-layer residues as those interacting with the first-layer residues and within 5 Å of first-layer residues; third-layer residues are those interacting with second-layer residues and within 5 Å of second-layer residues.

## Materials and methods

### Theoretical functional site prediction and layer analysis

One subunit of apo OTC from *E*. *coli* (PDB ID 1AKM, chain A; UniProt #P04391) [6] was pre-processed in YASARA [39] to add missing atoms. POOL [29, 32] calculations included input features from THEMATICS, INTREPID [40] and the structure-only version of ConCavity [38]. For present purposes, residues with normalized POOL scores greater than or equal to 0.01 [23] are predicted to be important for catalysis. Residues in direct contact with the substrates, ORN or CP, are considered to be first-layer residues. Second-layer residues are residues that are in contact with first-layer residues and within 5 Å of first-layer residues. Residues in direct contact with transition state analogues in the complex [41] were determined with the Ligand-Protein Contacts (LPC) server [42]. Further investigation into catalytic roles of POOL-predicted residues was conducted by docking CP and ORN into OTC (PDB # 1DUV) with Glide in Schrödinger Release 2016–2: Maestro (Schrödinger, LLC, New York, NY, 2017). The substrate positions were checked by aligning the protein with huOTC, with CP and L-norvaline, an ORN analogue, bound (PDB # 1C9Y). The substrates from our docked structure overlaid closely with the corresponding ligands in the huOTC structure. Residue contacts with docked CP and ORN and within layers were identified with Schrödinger and PoseView [43].

### Site-directed mutagenesis

A pCA24N plasmid expressing His-tagged OTC from *E*. *coli argI* gene was obtained from ASKA [44]. Variants were prepared with a Quikchange site-directed mutagenesis kit (Agilent) or by two-stage PCR with Q5 enzyme (New England Biolabs) using mutagenic DNA primers from Eurofins Operon and verified by DNA sequencing (Eton Bioscience, Charlestown, MA).

### Protein expression and purification

WT OTC and variants were expressed in BL21 (DE3) Tuner cells (Novagen) in 1 L of Luria broth with 25 μg/mL chloramphenicol at 37°C. Cells were grown to OD_600_ 0.6–0.8 and protein expression was induced with 0.5 mM isopropyl β-D-1-thiogalactopyranoside (IPTG) at 30°C for 4 h and harvested by centrifugation at 6000 x g for 10 min. The pellets were stored at -80°C until purification. Cell pellets were sonicated in equilibration buffer (50 mM HEPES, pH 8, 500 mM NaCl, 10 mM imidazole, 1 mM DTT) and treated with DNase I and lysozyme on ice for 1 h, followed by centrifugation at 14,000 x g to clear the lysate. The supernatant was loaded onto a 5 mL HisTrap FF column (GE Healthcare) with equilibration buffer and eluted with elution buffer containing 50 mM HEPES, pH 8, 500 mM NaCl, 500 mM imidazole, 1 mM DTT over a linear gradient of 10–500 mM imidazole. Elution fractions were concentrated to ~4 mL with 10 kDa MWCO Vivaspin 6 (Vivaproducts) concentrators at 7000 x g and loaded onto a HiLoad Superdex 200 26/60 size exclusion column (GE Healthcare) pre-equilibrated with 50 mM HEPES, pH 8, 200 mM NaCl, 2 mM DTT. Protein concentrations were determined by Bradford assay and proteins were stored in single-use aliquots at -80°C, under which conditions the proteins were stable for months. Proteins stored at -20°C and 4°C lost activity in less than one month.

### Kinetics assay

An optimized OTC kinetics assay measured citrulline (Sigma Life Science) production as described by Rahmatullah and Boyde (Fig 1) [45]. Detection of citrulline for this discontinuous assay is based on the Fearon method, in which the reaction between ketone diacetylmonoxime and citrulline, in the presence of strong acid, undergoes oxidation to produce an orange or pink pigment, suggested to be a pyrimidine ring derivative [14]. Ferric (III) chloride was also used to accelerate pigment formation [12]. Product turnover was determined with a 0.004–1 mM L-citrulline (Sigma Life Science) standard curve, which was within the linear range (typical R^2^ = 0.99). Reactions were carried out at 20°C ± 2°C in 50 mM Tris, pH 8.5, buffer; note that a consistent amount of enzyme was used such that reactions contained a final concentration of 20 mM NaCl. Reactions were initiated with 5–332 nM enzyme in Masterblock 96-well 1-mL plates (Greiner Bio-one) in which 100 μL reaction mixtures were quenched at 0.25–8 min in 500 μL freshly prepared color reagent containing 2:1 ratio of solutions #1 and #2. Solution #1 contained 250 mg iron (III) chloride hexahydrate (Alfa Aesar), 250 mL 99% sulfuric acid (BDH), 200 mL 85% phosphoric acid (BDH), 550 mL MilliQ water and solution #2 contained 2.5 g 2,3-butadione monoxime (diacetyl monoxime) (Sigma Life Science) in 500 mL MilliQ water. Thiosemicarbazide (TSC, Acros Organics), which acts as a color stabilizer [13, 45, 46], was added prior to use (1 mg TSC per 10 mL of solution #2) and the color reagent was used within 1 h of preparation. Solutions #1 and #2 were stored in amber bottles at 4°C for up to 2 and 1 month, respectively. Ornithine (Sigma Life Science) was varied from 0.05–48 mM (with carbamoyl phosphate held at 5 mM) and carbamoyl phosphate (Sigma Life Science) varied from 0.04–16 mM (with ornithine held at 3 mM or 60 mM for variants with *K*_M_ values that exceeded 3 mM ORN) [2, 3]. Holes in the four corners of the Masterblock 96-well plates allowed for even submersion of plates during the 5-min incubation in a 100°C water bath for color development. Plates were then cooled in a 13–15°C water bath, 200 μL aliquots were transferred to flat-bottom polystyrene 96-well microplates (Greiner Bio-One), and absorbance was read at 530 nM with a Biotek Synergy HT Plate Reader and Gen5 version 1.11 software. Linear initial rate data were fit to the Michaelis-Menten equation using GraphPad Prism 5.02 for Windows (GraphPad Software, San Diego California USA), to determine *K*_M_ and *V*_max_. Further calculations and averages of *k*_cat_ and *k*_cat_/*K*_M_ from at least three independent experiments were determined in Microsoft Excel®. Errors reported are the standard deviation.

### Thermal shift assay

Melting temperatures of WT OTC and variants were determined with a Bio-Rad C1000 Touch Thermal Cycler (CFX96 Real-Time System) and Bio-Rad CFX Manager 3.1.1517.0823 software that measures fluorescence due to Sypro Orange binding to hydrophobic regions of the protein as the temperature is increased from 4°C to 100°C in 0.2°C increments with a 10-sec dwell time [47, 48]. Samples of wild-type OTC and variants, analyzed in triplicate, were prepared in 96-well plates containing 50 mM HEPES, pH 8, 200 mM NaCl, 2 mM DTT, 10x Sypro Orange (Invitrogen) and 10 μM enzyme alone, enzyme with 1 mM carbamoyl phosphate, enzyme with 10 mM ornithine or enzyme with 10 mM citrulline. Melting temperatures (T_m_) were obtained from the software as the highest peak of a first derivative plot of the negative rate change in relative fluorescence (RFU) vs. temperature (T).

### Fluorescence binding assay

Ligand binding can be monitored by fluorescence of Trp125, Trp233, Trp192 and Trp243, with Trp125 and Trp233 contributing approximately 80% of the total fluorescence of OTC [49]. This method was applied to probe the effects of mutations at POOL-predicted positions on binding. Mixtures of 2 μM OTC without or with 5 mM CP, 50 mM ORN, or 50 mM CIT were prepared in 50 mM Tris, pH 8.49 at 22°C, incubated for 1 min at room temperature, excited at 278 nm and emission was monitored at 300–400 nm. Mixtures were scanned three times and carried out in triplicate. Samples were read in a sub micro fluorometer quartz cell (Starna, 16.50F-Q-10/Z20) with a Cary Varian Eclipse Fluorimeter. Subtracted spectra were obtained by normalizing all spectra with respect to apo OTC, then subtracting the spectrum of the mixture from the spectra of apo OTC, buffer, and respective substrates alone.

### MCCE electrostatic calculations

MCCE (Multi-Conformation Continuum Electrostatics) was used to predict p*K*_a_ values of designated residues, coupled protonation equilibria, and coupled conformational states between residues [50, 51]. MCCE computes the protonation behavior of the amino acids in a protein structure through the combination of continuum electrostatics and molecular mechanics, specifically allowing the residue side chains to move. Input structures were built using the homology modeling module in the YASARA [52] suite of programs. A total of six or seven models were made from the top five templates, with up to five alignments per template. Each final model was built using the anabolic OTC trimer from *E*. *coli* (PDB # 1DUV) [41] as the template with the exception of Y160S (hybrid model based on PDB # 1DUV and 2OTC [7]) and R57A (hybrid model based on PDB # 1DUV and 4H31, anabolic ornithine carbamoyltransferase from *Vibrio vulnificus*, complexed with carbamoyl phosphate and L-norvaline); as YASARA automatically chooses the most similar structure for modeling, some models were built with different starting structures.

Homology models of WT OTC and OTC D140N, Y160F, Y160S, H272L, H272N, C273A, E299D, E299Q, R57A (+), S61A (-), and Q104L (-), were processed to add missing atoms and the transition state analog substrate was removed in YASARA. The minimization option in YASARA was used to minimize structures with the Yamber 3 force field, simulation box with 12 Å margin around all atoms, pH 8.5, 0.9% NaCl, and 1.0 g/mL water density. The minimized structures were submitted to the PDB2PQR online server [53] with CHARMM force field and protonation states at pH 8.5 assigned with PROPKA. Structures were next converted to PSF files in VMD with the automatic PSF builder module and topo_all_22 topology file [54]. The PSF files were further minimized in Gromacs for 5 nsec. The final structures were submitted to MCCE for electrostatic calculations. Coupled protonation equilibria and coupled rotamer states between residues were obtained with the getinfo option in MCCE.

### Size-exclusion chromatography-small angle X-ray scattering data collection

In-line Size-Exclusion-Chromatography-coupled SAXS (SEC-SAXS) data were collected at the G1 beamline at Cornell High Energy Synchrotron Source using a Superdex 200 5/150 GL column. WT OTC and variants at 4.3–5.5 mg/mL and concentrated stocks of CP and ORN were transported on dry ice and thawed on ice before use. Binary complexes were also prepared with enzyme incubated with saturating/final concentrations of 5 mM CP and 10 mM ORN for 20 min, on ice, before samples were loaded onto the SEC column. Solution scattering data were captured continuously every 2 sec for 2000 frames for the duration of the SEC run. Data were collected from the maxima of peak(s) representing the protein of interest, avoiding aggregates. Data for the running buffer were collected as a blank that was subtracted from the protein data to remove any scattering that was the result of the background buffer.

### SAXS data analysis

Initial data processing was undertaken automatically using the RAW software at the beamline [55, 56]. Further processing using the ATSAS suite (version 2.7.1-http://www.embl-hamburg.de/biosaxs/software.html) was done using PRIMUS [57] for initial radius of gyration (R_g_) calculations, pair-distribution plots, and Kratky plot analysis; GNOM [58] was used for normalization of SAXS data and GASBOR [59] for generating *ab initio* models. Figures were produced using CHIMERA [60] followed by superimposition of envelopes.

### Reconstruction of molecular envelopes

Programs within the ATSAS suite [61] were used to determine the three-dimensional molecular envelopes for WT OTC and variants in the apo and ligand-bound forms using the solution x-ray scattering data. The GNOM program [58] was used to evaluate the pair distribution plot using an indirect Fourier transform from the x-ray solution scattering data with an r_max_ = ~120 Å. Due to the suggested structural rearrangement, some variants had a larger r_max_ value. The GASBOR program [59] was used to generate three-dimensional models of connected beads to fit the GNOM data, with the number of beads set approximately equal to the total number of amino acids in the ornithine transcarbamoylase construct (999 residues). In order to assess the uniqueness of these solutions, 10 bead models were generated with and without three-fold symmetry. The final χ^2^ values of these models ranged from 0.99 to 1.5.

The independent bead models were aligned for averaging with the SUPCOMB program [62]. The ten aligned models were averaged to provide a three-dimensional reconstruction of the shape of OTC with a partial specific volume set to the expected value from the OTC sequence. Results were then visualized using the graphics of Chimera [60].

### Explicit-solvent simulations

All explicit-solvent simulations were performed with Gromacs v4.6.5 [63, 64] using the CHARMM 27 force field [65, 66]. In addition to simulating the wild-type protein, the following variants were also simulated: C273A, D140N, E299D, E299Q, H272L, H272N, Q104L, R57A, S61A, Y160F, Y160S, Y229F and Y229S. Each system was simulated according to the following protocol: The solute was placed in a box of dimensions 110 Å x 110 Å x 110 Å and then solvated using the TIP3P water model [67]. The system was subjected to steepest descent energy minimization, followed by conjugate gradient minimization, while position restraints were maintained on the solute. To equilibrate the density of water, each system was simulated with the NVT ensemble for 2 ns, where temperature was held at 293 K via the Nose-Hoover thermostat [68, 69], with position restraints placed on solute. This was followed by equilibration for 10 ns under the NPT ensemble using the Parrinello-Rahman barostat [70–72], where the temperature was maintained at 293 K via the Nose-Hoover thermostat. Production simulations were then performed using the NPT ensemble for a minimum of 1 μsec, each.

### Simulations with structure-based models

An all-atom structure-based model was employed [73], where every non-hydrogen atom was explicitly represented. The force field was prepared using the SMOG 2 software package [74], with default all-atom parameters. In this model, all bond lengths, bond angles and dihedral angles are defined to have potential energy minima at their native value. In addition, non-bonded interactions between atoms that are in contact in the native configuration are defined by attractive 6–12 potentials, with minima corresponding to the distances found in the native configuration. Native contacts were defined based on the Shadow algorithm [75].

Simulations were performed using Gromacs v5.1.4 [63, 64]. The system was held at a constant temperature of 0.75 (reduced units). This temperature was used, since it has been shown to describe accurately the scale and distribution of structural fluctuations at 300 K [76]. In addition, the scale and distribution of the RMSF values were similar between explicit-solvent simulations at 293 K and the values obtained at 0.75 in the SMOG model. 10,000,000 timesteps of 0.002 reduced units were used for equilibration. Subsequently, the simulation was performed for an additional 118,000,000 timesteps. The RMSF values and principal components were based on the coordinates of all Cα atoms.

## Results

### POOL prediction of functionally important residues

POOL predicted 12 catalytically important residues for *E*. *coli* OTC with normalized POOL scores (Table 1) [23] above a 0.01 cut-off before the scores plateau and we focused on the top ten predicted residues. Six of the seven residues that have been identified previously to be catalytically important (Arg57, Arg106, His133, Asp231, Cys273, and Arg319) [7, 41] were among the top 12 POOL-predicted residues. Another known catalytic residue, Gln136 [7], was ranked twentieth (top 6%) by POOL. During catalysis, CP binds in a pocket where Arg106, Arg57, Thr56, Ser55, Thr58 and neighboring Gln82 stabilize the negative charge of the phosphonate moiety [2, 7, 41, 77]. The negative charge of the carbonyl oxygen atom is stabilized by side chains of His133 and Arg106 and by electrostatic interactions with Arg319 during nucleophilic attack [41]. In addition, protonated Arg57 has been shown to be important for sequential binding of CP followed by ORN, for selection and binding of the correct l-ORN enantiomer and for a CP-induced conformational change that controls product turnover in the forward reaction [2, 9, 78–80].

**Table 1:** POOL prediction of catalytically important residues.

POOL Rank	Residue	Layer	Distance from substrate (Å)	Normalized POOL Score
1	Cys273	1^st^	3.0^a^	1.00
2	His272	2^nd^	5.9^b^	0.49
3	Arg319	1^st^	2.7^a^	0.32
4	Tyr229	2^nd^	7.0^b^	0.32
5	Asp231	1^st^	2.6^b^	0.31
6	Asp140	2^nd^	6.9^a^	0.16
7	Glu299	3^rd^	8.7^b^	0.12
8	Tyr160	2^nd^	5.1^b^	0.05
9	Arg106	1^st^	2.6^a^	0.04
10	His133	1^st^	3.0^a^	0.02
11	His321	3^rd^	12.3^a^	0.01
12	Arg57 (+)	1^st^	2.9^a^	0.01
20	Gln136	1^st^	3.0^a^	0.00
93	Ser61 (-)	2^nd^	6.5^a^	0.00
170	Gln104 (-)	3^rd^	9.0^a^	0.00

^a^Distance to CP

^b^Distance to ORN

**Fig 2:**
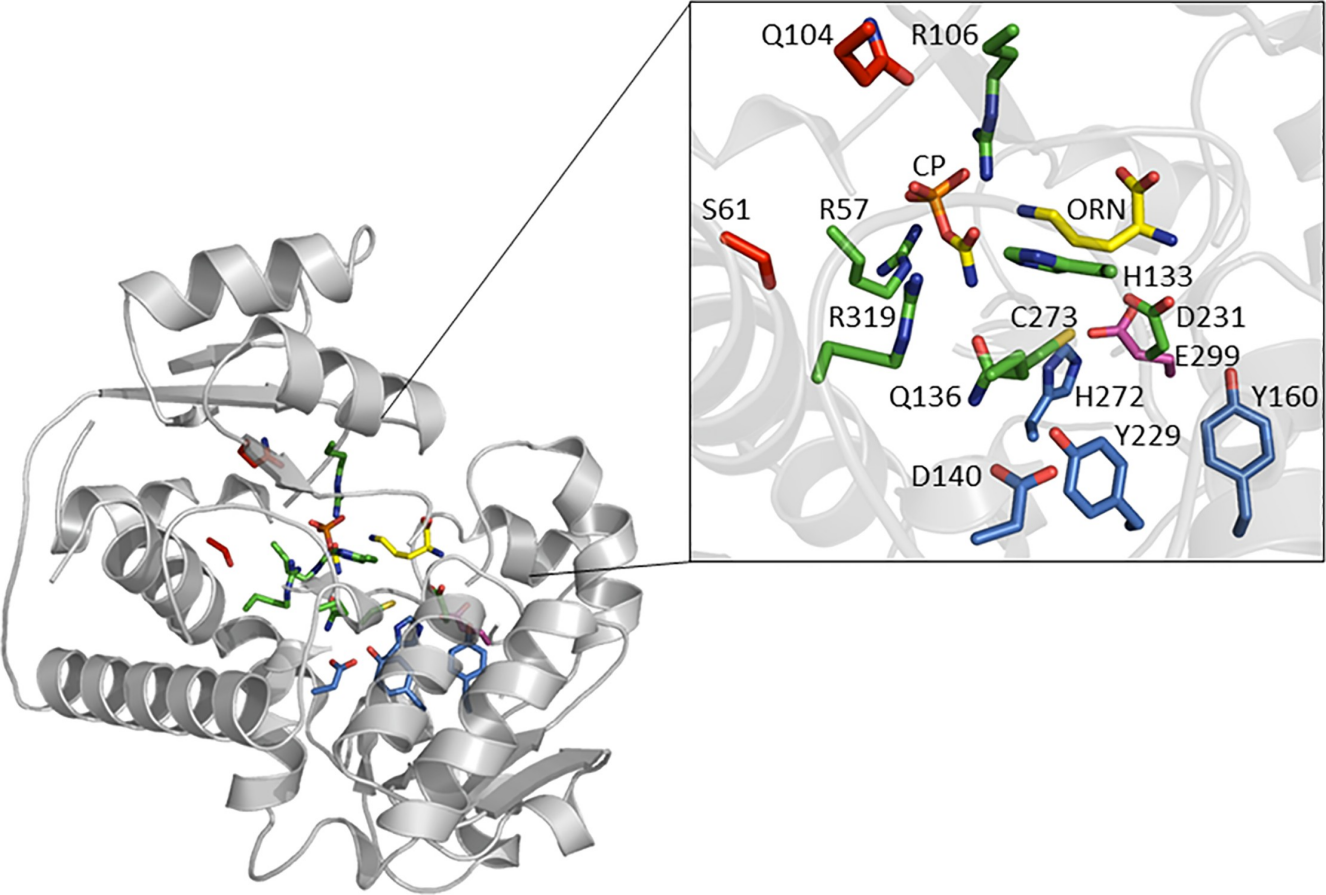
OTC active site and POOL-predicted residues (*E*. *coli*). A subunit of OTC (PDB # 1DUV) [41] is shown here with CP and ORN docked (shown in yellow and elemental coloring). Residues Arg57, Arg106, His133, Gln136, Glu231, Cys273, and Arg319 have been reported to be important for catalysis (shown in green). POOL predicts Asp140, Tyr229, His272 and Tyr160 in the second layer (shown in blue) and Glu299 in the third layer (shown in magenta) to be important for catalysis. Negative controls Ser61 and Gln104 (shown in red) had low POOL scores and high ConSurf [83] conservation scores.

Following the conformational change, the deprotonated thiol group of Cys273 has been reported to bind the correct ORN enantiomer by forming a salt bridge with the δ-amino moiety of ORN [6, 79, 81]. We note that the deprotonated state of the thiol group of Cys273 can be populated at neutral pH because this protonation equilibrium is coupled to that of other residues, including the nearby Tyr229, thereby expanding the buffer range of Cys273. Cys273 is at the binding site for ORN, however, it is not the only residue that can form an anion that is in position to assist with ORN binding [79]. Interactions between the positively charged α-amino group of ORN and the carboxylate group of Asp231 provide additional binding energy for ORN [6, 7, 41, 77, 79, 82]. Crystal structures also show the δ-amino group of ORN to hydrogen bond to the backbone carbonyl oxygen atom of Leu274 that is part of a conserved loop (His272-Pro275) [7]. In addition, the α-amino group of ORN hydrogen bonds to the carbonyl oxygen atom of Asn167. The carboxyl oxygen atom of ORN is hydrogen bonded to the backbone nitrogen atom of Met236 [41]. The five remaining residues with high POOL rank and located in the second- and third-layers of the OTC active site, up to 15 Å away from the substrates, are Asp140, Tyr160, His272, Tyr229 and Glu299 (Fig 2). These residues are further discussed below.

We constructed the following OTC variants harboring mutations of POOL-predicted residues to test their effects on activity: R57A, D140N, Y160F, Y160S, Y229S, Y229F, D231A, H272L, H272N, C273A, E299Q, and E299D. These substitutions were generally chosen to be as conservative as possible. The Asp to Asn and Glu to Gln substitutions eliminate the negative charge of the carboxylate group but preserve the size and polarity of the side chain. The Glu to Asp substitution tests the effects of distance to the charged carboxylate group. The Tyr to Phe and Tyr to Ser substitutions test the relative importance of the aromatic ring and the phenolic–OH group of Tyr. There is no truly conservative mutation for His; Asn and Leu are roughly of similar size, the former being polar and the latter non-polar. Previously, site-directed mutagenesis and kinetics study of the OTC variant R57G resulted in a turnover rate 21,000-fold lower than that of wild-type (WT) OTC [2] and mutation of the spatially aligned residue in huOTC, Arg92, is assocated with OTCD [84]. Therefore, R57A is a positive control (+) for this study as it is likely to result in decreased activity. The S61A and Q104L mutations are negative controls (-) that are located in the second- and third-layer, respectively. The negative controls have high sequence conservation scores, as determined by ConSurf [83], and low POOL scores (Table 1). The POOL-predicted first-layer residues Arg57, Arg106, His133, Asp231, Cys273 and Arg319 are spatially aligned with huOTC Arg92, Arg141, His168, Asp263, Cys303 and Arg330, respectively. Four of the POOL-predicted distal residues, Asp140, Tyr160, His272, and Glu299, are spatially aligned with huOTC Asp175, Trp193, His302, and Glu310, respectively. Mutations of these residues are known to occur in patients with OTCD [15, 85–91].

### Mutation of POOL-predicted residues impacts catalysis and stability

Results of steady-state kinetics assays for WT OTC and its variants, with respect to CP and to ORN, are shown in Tables 2 and 3, respectively. Variants D231A, H272L, E299Q, and R57A (+) showed 450-, 310-, 110-, and 57-fold decreases in catalytic efficiency (*k*_cat_/*K*_M_), respectively, with respect to CP. Variants D140N, Y160F, Y160S, Y229F, Y229S, H272N, C273A, E299D, S61A (-), and Q104L (-) had catalytic efficiencies that were decreased less than ten-fold from that of WT OTC with respect to CP. Variants D140N, Y160S, D231A, H272L, E299Q, R57A (+) and showed 28-, 14-, 580-, 120-, 51-, and 44-fold decreases in catalytic efficiency (*k*_cat_/*K*_m_), respectively, with respect to ORN. Variants Y160F, Y229F, Y229S, H272N, C273A, E299D, S61A (-) and Q104L (-) had decreases in catalytic efficiencies of ten-fold or less than that of WT OTC with respect to ORN. Variants D231A, H272L, E299Q, and R57A (+) had substantial decreases in relative catalytic efficiencies with respect to both CP and ORN.

**Table 2:** Steady-state kinetic parameters of OTC and its variants with respect to carbamoyl phosphate.

	Layer	*K*_M_ (mM)^a^	*k*_cat_ (s^-1^)^a^	*k*_cat_/*K*_M_ (x10^4^ M^-1^s^-1^)^a^	Relative *k*_cat_	Relative *k*_cat_/*K*_M_	Fold-decrease *k*_cat_/*K*_M_
Wild-type	---	0.27 ± 0.034	410 ± 20	150 ± 13	1.0	1.0	1.0
D140N	2^nd^	1.4 ± 0.45	360 ± 59	29 ± 9.0	0.89	0.19	5.4
Y160F	2^nd^	0.53 ± 0.12	120 ± 18	23 ± 3.9	0.30	0.15	6.7
Y160S	2^nd^	0.30 ± 0.063	300 ± 50	100 ± 14	0.74	0.65	1.5
Y229F	2^nd^	0.33 ± 0.11	150 ± 58	44 ± 11	0.36	0.28	3.5
Y229S	2^nd^	0.32 ± 0.025	290 ± 120	92 ± 45	0.72	0.60	1.7
D231A	1^st^	0.15 ± 0.042	0.48 ± 0.10	0.34 ± 0.13	0.0012	0.0022	450
H272L	2^nd^	0.93 ± 0.58	4.2 ± 1.8	0.50 ± 0.11	0.010	0.0032	310
H272N	2^nd^	0.50 ± 0.16	180 ± 65	36 ± 1.5	0.45	0.24	4.2
C273A	1^st^	0.26 ± 0.063	140 ± 43	56 ± 15	0.35	0.36	2.8
E299D	3^rd^	0.22 ± 0.083	120 ± 45	62 ± 34	0.29	0.40	2.5
E299Q	3^rd^	0.34 ± 0.11	4.6 ± 1.0	1.4 ± 0.33	0.011	0.0093	110
R57A (+)	1^st^	0.28 ± 0.027	7.5 ± 1.8	2.7 ± 0.73	0.018	0.018	57
S61A (-)	2^nd^	0.30 ± 0.036	74 ± 40	25 ± 15	0.18	0.16	6.1
Q104L (-)	3^rd^	0.40 ± 0.30	88 ± 36	26 ± 7.5	0.22	0.17	5.9

^a^Errors reported are standard deviation. The three parameters *k*_cat_, *K*_M_, and *k*_cat_/*K*_M_ were each determined from at least three independent experiments; thus *k*_cat_/*K*_M_ does not always equal exactly the result of *k*_cat_(avg)/*K*_M_(avg).

**Table 3:** Steady-state kinetic parameters of OTC and its variants with respect to ornithine.

	Layer	*K*_M_ (mM)^a^	*k*_cat_ (s^-1^)^a^	*k*_cat_/*K*_M_ (x10^4^ M^-1^s^-1^)^a^	Relative *k*_cat_	Relative *k*_cat_/*K*_M_	Fold-decrease *k*_cat_/*K*_M_
Wild-type	---	0.53 ± 0.055	440 ± 9.2	83 ± 7.8	1.0	1.0	1.0
D140N	2^nd^	17 ± 5.9	460 ± 154	3.0 ± 1.7	1.1	0.036	28
Y160F	2^nd^	1.0 ± 0.16	110 ± 34	10 ± 2.0	0.24	0.12	8.2
Y160S	2^nd^	7.3 ± 2.0	410 ± 160	6.1 ± 3.2	0.94	0.073	14
Y229F	2^nd^	0.45 ± 0.11	130 ± 46	31 ± 13	0.30	0.37	2.7
Y229S	2^nd^	0.61 ± 0.15	350 ± 170	59 ± 34	0.78	0.71	1.4
D231A	1^st^	0.59 ± 0.33	0.70 ± 0.050	0.14 ± 0.067	0.0016	0.0017	580
H272L	2^nd^	0.68 ± 0.28	4.3 ± 0.65	0.71 ± 0.31	0.010	0.0085	120
H272N	2^nd^	0.40 ± 0.065	95 ± 45	24 ± 14	0.21	0.29	3.4
C273A	1^st^	0.73 ± 0.35	170 ± 56	26 ± 9.3	0.39	0.31	3.2
E299D	3^rd^	0.90 ± 0.17	170 ± 36	20 ± 5.9	0.39	0.24	4.2
E299Q	3^rd^	0.33 ± 0.10	5.1 ± 1.0	1.6 ± 0.49	0.012	0.020	51
R57A (+)	1^st^	0.44 ± 0.092	8.2 ± 1.1	1.9 ± 0.39	0.019	0.023	44
S61A (-)	2^nd^	0.53 ± 0.18	240 ± 75	46 ± 6.4	0.54	0.56	1.8
Q104L (-)	3rd	0.81 ± 0.23	52 ± 51	8.0 ± 3.3	0.12	0.096	10

^a^Errors reported are standard deviation. The three parameters *k*_cat_, *K*_M_, and *k*_cat_/*K*_M_ were each determined from at least three independent experiments; thus *k*_cat_/*K*_M_ does not always equal exactly the result of *k*_cat_(avg)/*K*_M_(avg).

**Fig 3:**
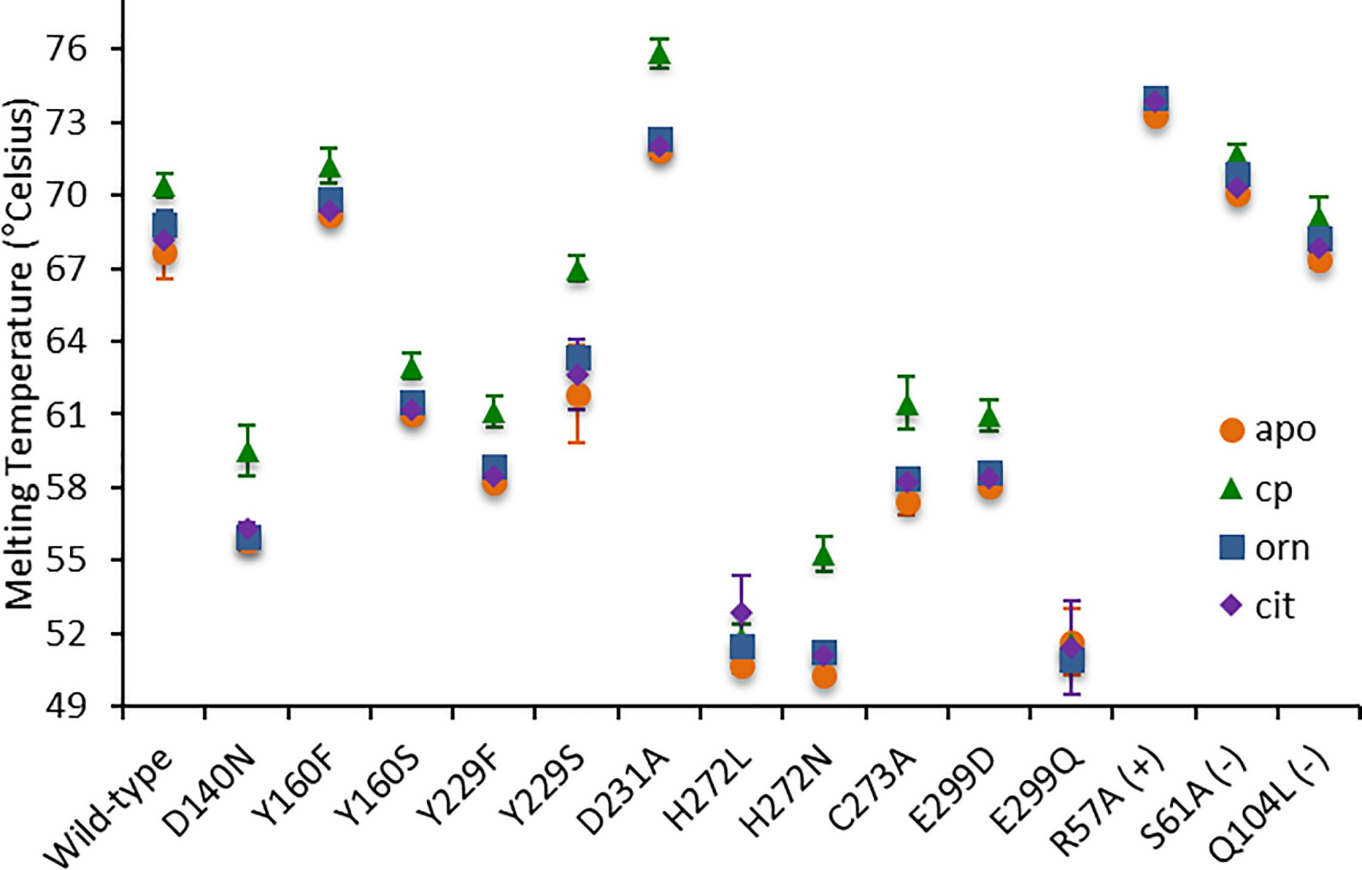
Thermal shift assay with respect to apo enzyme (orange), enzyme+CP (green), enzyme+ORN (cyan) and enzyme+CIT (yellow). Addition of CP resulted in a higher T_m_ of WT OTC and variants, compared to apo, enzyme+ORN, or enzyme+CIT, except H272L, E299Q and R57A.

To determine the effect of the mutations on protein stability, thermofluor (thermal shift) assays were carried out with WT and OTC variants in the apo state and with CP, ORN, or CIT (Table 4). The melting temperature (T_m_) for apo WT OTC is found to be 68 ± 1.1°C. Y160F, S61A and Q104L have T_m_ values within 2.0°C of WT, and R57A (+), and D231A have T_m_ values 4.0–5.0°C higher than WT. Remaining variants, D140N, Y160S, Y229F, Y229S, H272L, H272N, C273A, E299D and E299Q have T_m_s ranging from 50–62°C. We observed a trend of increasing T_m_ in the presence of CP compared to apo enzyme, enzyme + ORN, and enzyme + CIT, except for H272L, E299Q and R57A (+) control (Fig 3). Addition of CP to H272L, E299Q and R57A (+) did not increase the T_m_ of these OTC variants. The OTC variants H272L, H272N, and E299Q were the least stable, with T_m_s reduced more than 15°C relative to WT OTC. Catalytic efficiency with respect to ORN is only weakly correlated with T_m_ (R = +0.32). Although variants that are not stabilized by CP binding do have low catalytic efficiency with respect to CP, we did not observe overall any significant correlation between variant T_m_ and catalytic activity (R = +0.10), indicating that the effects of the mutation on activity are not solely due to changes in protein stability. We note that all variants that are not stabilized by CP (Fig 3) also show dramatically reduced activity with respect to CP (Table 2). The lack of increased Tm in the presence of CP for H272L, E299Q and the R57A (+) positive control also correlates with the lack of substrate-induced changes in fluorescence, as discussed below.

**Table 4:** Melting temperatures (T_m_) of WT OTC and variants.

	apo	enzyme +cp	enzyme +orn	enzyme +cit
Wild-type	68 ± 1.1	70 ± 0.5	69 ± 0.6	68 ± 0.8
D140N	56 ± 0.3	60 ± 1.0	56 ± 0.3	56 ± 0.2
Y160S	61 ± 0.0	63 ± 0.5	62 ± 0.1	61 ± 0.0
Y160F	69 ± 0.2	71 ± 0.7	70 ± 0.2	69 ± 0.0
Y229F	58 ± 0.1	61 ± 0.6	59 ± 0.2	59 ± 0.1
Y229S	62 ± 2.0	67 ± 0.5	63 ± 0.8	63 ± 1.4
D231A	72 ± 0.3	76 ± 0.6	72 ± 0.3	72 ± 0.5
H272L	51 ± 0.3	52 ± 0.6	52 ± 0.1	53 ± 1.5
H272N	50 ± 0.1	55 ± 0.8	51 ± 0.1	51 ± 0.1
C273A	57 ± 0.5	62 ± 1.1	58 ± 0.2	58 ± 0.2
E299D	58 ± 0.2	61 ± 0.6	59 ± 0.2	58 ± 0.0
E299Q	52 ± 1.3	52 ± 0.2	51 ± 0.5	51 ± 1.9
R57A (+)	73 ± 0.3	74 ± 0.0	74 ± 0.0	74 ± 0.3
S61A (-)	70 ± 0.2	72 ± 0.4	71 ± 0.1	70 ± 0.1
Q104L (-)	67 ±0.3	69 ± 0.8	68 ± 0.2	68 ± 0.2

### Binding assay reveals altered fluorescence difference spectra of OTC variants for CP binding

**Fig 4:**
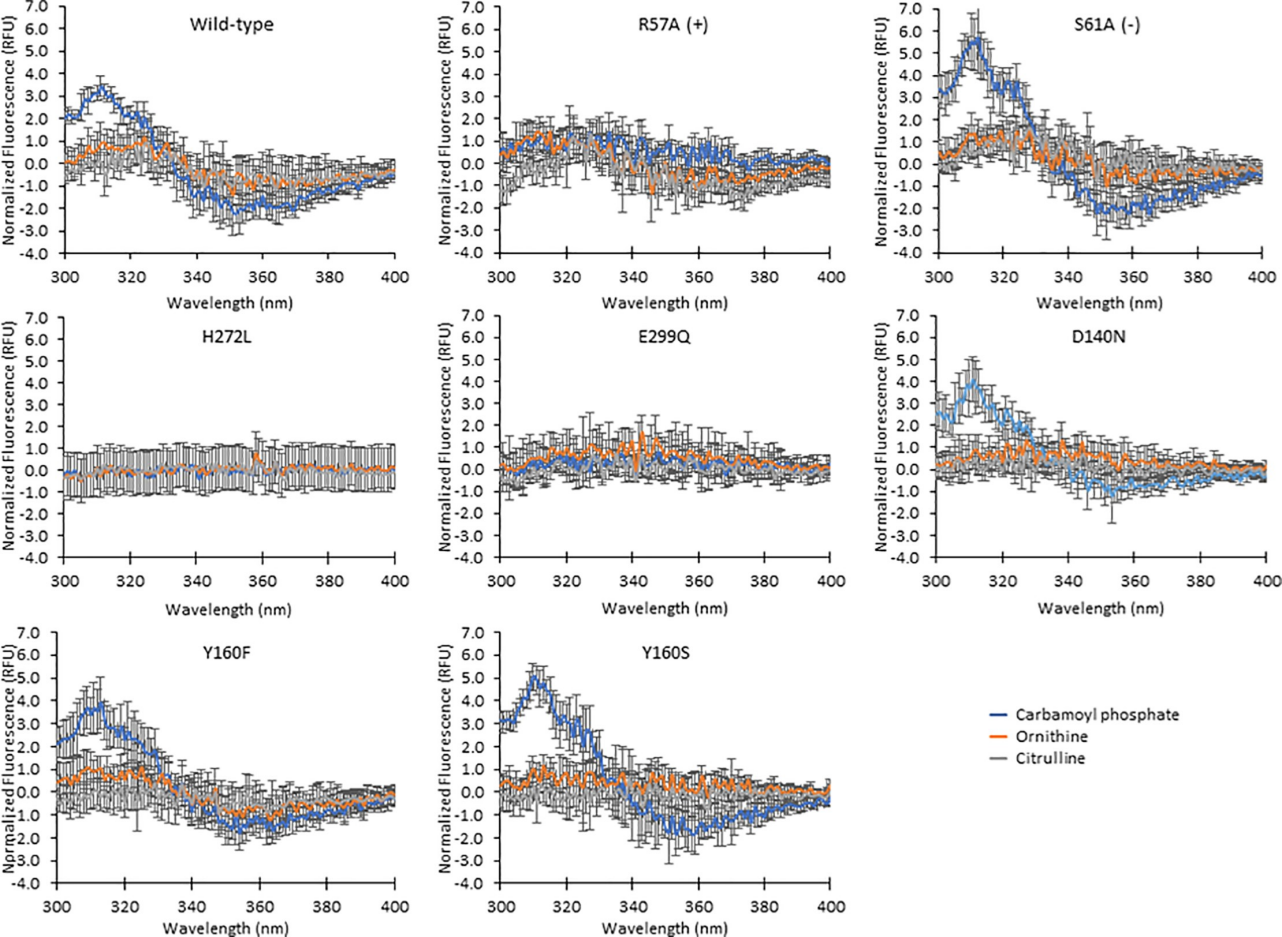
Tryptophan fluorescence binding assay with respect to protein +CP, +ORN, and +CIT. Difference fluorescence spectra were obtained by normalizing spectra to apo then buffer, apo and respective substrate spectra were subtracted from the spectrum of the mixture of protein with substrate. Data are reported as the average of three trials and the error bars indicate the standard deviation.

We assessed binding of substrates to WT OTC and selected variants by intrinsic tryptophan fluorescence spectroscopy [49]. WT OTC difference fluorescence spectra resulted in a shift in the peak at 310 nm in the presence of CP relative to that of WT OTC alone (Fig 4). Difference spectra for WT with ORN and WT with CIT resulted in a minor, broad peak at 300–340 nm. Additional variants S61A (-) control, D140N, Y160F and Y160S had similar difference spectra to WT, suggesting binding similar to that of WT. OTC variants H272L, E299Q and R57A (+) control did not have a difference peak at 310 nm in the presence of CP, possibly suggesting that mutations at these positions may cause somewhat diminished CP binding. However, we note that Glu299 in the crystal structure is about 4 Å away from Trp233 and about 4 Å away from Trp243 and in turn, His272 interacts with Glu299; therefore changes in the difference spectra of the variants E299Q and H272L may arise from the altered environment around Trp233 and Trp243.

### Calculation of residue-residue coupling energies

**Fig 5:**
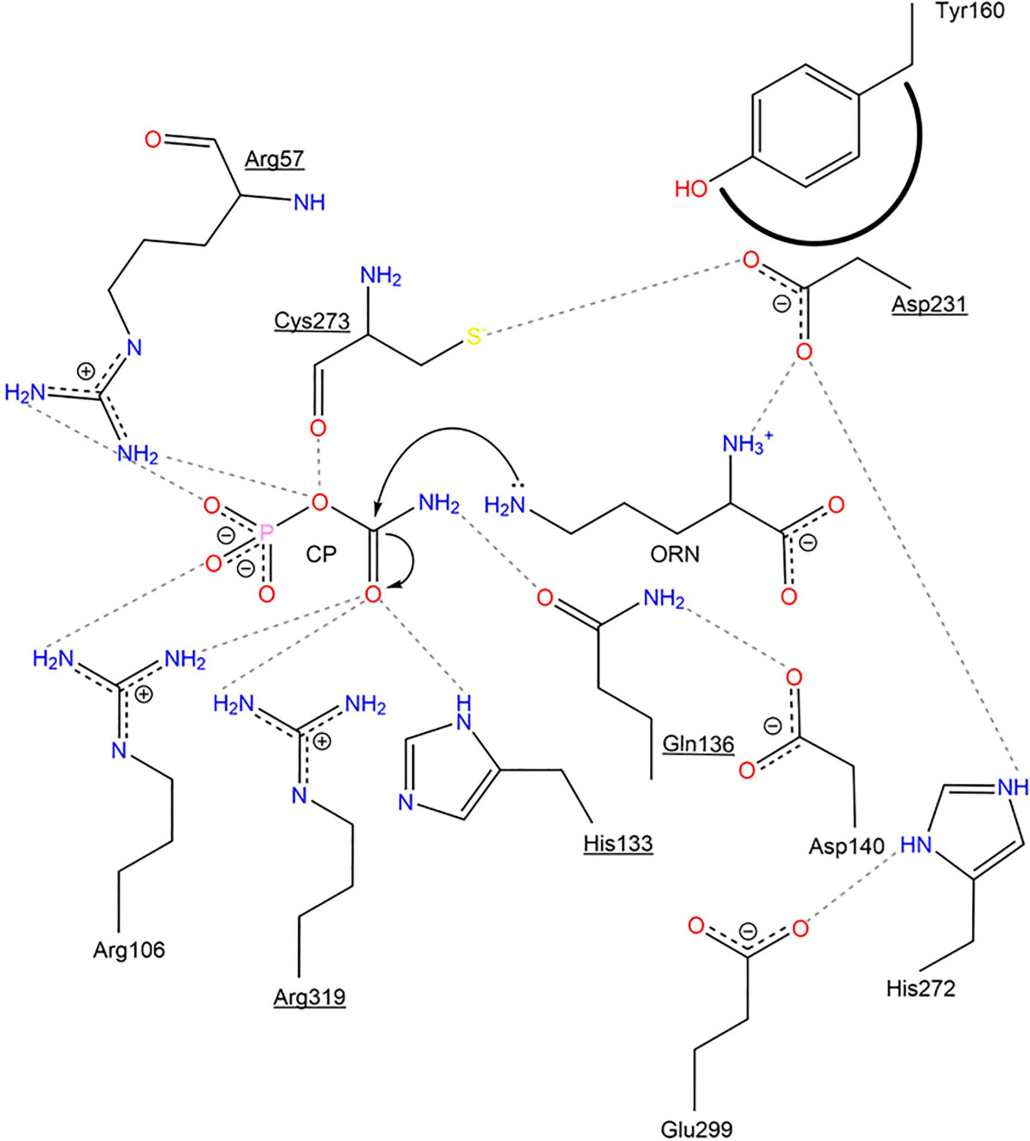
2D representation of OTC active site. Arg57, Arg106, His133, Gln136, Asp231, Cys273 and Arg319, underlined, are catalytic residues that are in direct contact with CP and ORN. POOL-predicted remote residues in the second-layer are Asp140, Tyr160, Tyr229 and His272. Glu299, located in the third-layer, is also predicted by POOL to play an important role in catalysis. Hydrogen bonds are represented as dotted lines and hydrophobic interactions are represented by bold arched lines.

OTC D140N has a catalytic efficiency 28-fold less than WT with respect to ORN and 5.4-fold less with respect to CP. MCCE results show that the protonation equilibrium of D140 is coupled to those of Arg57, Tyr229 and Cys273; this coupling is lost in the variant D140N. OTC E299Q, H272L and R57A (+) all show substantial loss of catalytic efficiency relative to WT with respect to both CP and ORN. Because His272 and Glu299 can form a salt bridge (Fig 5) [6, 10], the protonation equilibrium of Glu299 is coupled both electrostatically and conformationally with His272; this coupling is lost in the E299Q variant. The positive control R57A (+) is predicted to lose coupling of the protonation equilibrium of Arg57 with Asp87, His133, Asp140, His272, Cys273, and Arg319.

### SEC-SAXS reveals conformational changes of certain variants

**Fig 6:**
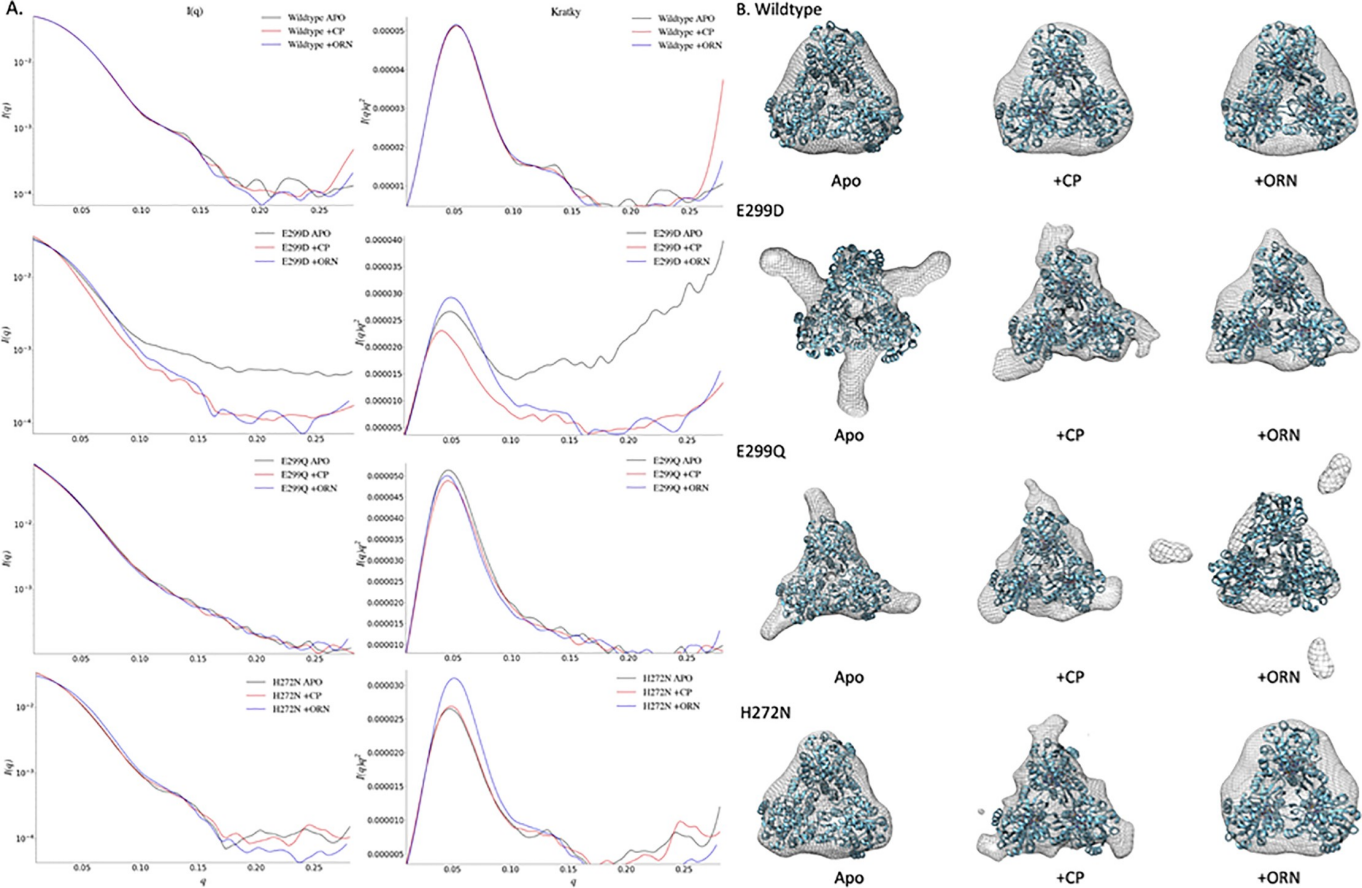
SAXS analysis of OTC and variants. A. SAXS patterns after background subtraction of WT OTC and E299D, E299Q, and H272N variants with the addition of substrates along with the Kratky plots. Changes in the scattering patterns are due to substrate binding. Kratky plots show that WT OTC is properly folded, however other variants such as E299D in the apo state show a partially unfolded structure. The Kratky plots of E299Q with and without substrate are similar, in which a portion of the structure appears to be extended or flexible. B. Three-dimensional shape envelopes were constructed based on these SAXS patterns of WT OTC, and OTC E299D, E299Q, and H272N with and without substrate. Crystal structure is embedded in the reconstruction for reference. Shape reconstructions suggests a portion of the OTC structure to be flexible in some of these variants.

To gain additional insight into possible conformational perturbations of the variants, we carried out SEC-SAXS. Analysis of the apo WT OTC solution scattering curve corresponded to a soluble OTC trimer with a radius of gyration R_g_ of 33.3 Å (Table 5), which constitutes a small increase from the 31.2 Å that was predicted for the crystal structure of the 99-kD globular protein (PDB # 1AKM; UniProt # P04391) [6]. This difference in predicted and calculated R_g_ may be partially a result of the uncleaved 6x-His tag that was expressed with the enzymes. The solution scattering curve of WT OTC in the presence of CP had a R_g_ of 33.1 Å (Table 5, Fig 6). The R_g_ further contracts to 32.3 Å upon the addition of ORN. In both cases, the calculated R_g_ deviated by less than 3 Å from the predicted 30.2 Å calculated from the crystal structure of OTC with a substrate mimic bound (PDB # 1DUV; Uniprot # P04391) [41] and less than 2 Å from the value of 30.7 Å predicted from the structure with a different substrate mimic bound (PDB ID # 2OTC) [7]. The addition of these substrates shows clear differences in q in the range of 0.20–0.25 Å^-1^. Here q is the momentum transfer, which is related to the scattering angle by q = (4π sinθ)/λ, where 2θ is the scattering angle and λ is the x-ray wavelength. The range where differences are obvious is at the high end of the range of observed q, indicating that the differences correspond to alterations of relatively small parts of the protein structure. This indicates conformational differences between the binary complexes of OTC with CP and OTC with ORN.

**Table 5:** Radius of gyration (R_g_) of WT OTC and variants from SEC-SAXS. The R_g_ of WT OTC and variants were calculated for the apo state, with the addition of carbamoyl phosphate (CP), and with addition of ornithine (ORN).

Variant	Layer	Rg (apo, Å)	Rg (enzyme+CP, Å)	Rg (enzyme+ORN, Å)
WT	---	33.3	33.1	32.3
D140N	2^nd^	57.9	45.0	35.7
Y160F	2^nd^	34.9	35.1	34.5
Y160S	2^nd^	33.4	34.0	33.2
Y229F	2^nd^	33.6	34.6	33.3
Y229S	2^nd^	32.8	32.9	32.6
D231A	1^st^	33.1	33.1	33.1
H272L	2^nd^	40.7	42.0	-----
H272N	2^nd^	38.9	40.2	33.2
C273A	1^st^	41.0	39.1	34.1
E299D	3^rd^	39.6	44.5	35.8
E299Q	3^rd^	60.1	58.7	44.5
R57A (+)	1^st^	37.2	39.1	37.1
S61A (-)	2^nd^	33.3	33.1	32.0
Q104L (-)	3^rd^	33.1	32.8	32.5

Negative control variants, S61A and Q104L, had similar curves to WT with R_g_ of 33.3 Å and 33.1 Å, respectively. The addition of substrates changed the R_g_ anywhere between 0.3–1.0 Å, depending on the substrate present. In all cases, the features of the Kratky plot correspond to those expected for a well-folded protein in solution.

OTC E299D and E299Q, both variants with mutations to a POOL-predicted third-layer residue, show significant differences in the scattering patterns compared to WT OTC (Fig 6). The scattering patterns did not show signs of aggregation or inter-particle interaction. The R_g_ values for these two variants are significantly greater than that of WT; having R_g_ of 39.6 Å and 60.1 Å in the apo form, for E299D and E299Q, respectively. These R_g_ values indicate a substantial increase in molecular size, relative to that predicted from the crystal structure. The near doubling of R_g_ in the E299Q variant indicates a major structural reconfiguration. The features of the Kratky plots suggest that these apo states are partially or nearly-fully unfolded (Fig 6). When CP is added the R_g_s are 44.5 Å and 58.7 Å, respectively. Ornithine reduces the R_g_ values to 35.8 Å and 44.5 Å, respectively. Kratky plots show that the addition of CP and ORN to these variants results in well-folded structures (Fig 6). One possible explanation for the large increase in R_g_ is a flexible loop in the structure moving outward, causing an increase in R_g_ for these variants. In some variants such as OTC E299D, this loop could move back in when substrates are added and this is presumably why OTC E299D maintains catalytic activity.

Other variants with mutations to first- and second-layer residues, D140N, H272L, H272N, C273A, and R57A (+) also showed differences in the scattering patterns compared to WT OTC. These variants all have larger radii of gyration ranging from 4–25 Å greater than WT OTC. Unlike WT OTC, the addition of CP increases the R_g_ in both H272 variants and the R57A variant. Although these variants have larger R_g_ values, the Kratky plots show the proteins to be well-folded. The envelope for OTC R57A has a small structural change that is not seen with the addition of ORN. The H272L and H272N variants did not show significant structural changes in the apo form, relative to WT OTC. For OTC H272N, we observed a similar overall global structure to the apo state of WT OTC. However, upon the addition of CP, areas of the structure appeared to adopt a different conformation relative to WT OTC. These changes are consistent with the presence of a small flexible loop that moves outward; this change is not observed with the addition of ornithine. In the case of OTC H272L, only small fluctuations are seen in the apo state and with the addition of CP, relative to WT OTC. Unfortunately, we tried several times to collect data on H272L with the addition of ORN, but were not able to obtain the data due to the insolubility of the protein.

Three-dimensional envelopes of WT OTC were aligned with the corresponding PDB files, PDB # 1AKM [6] and PDB # 1DUV [41] (Uniprot # P04391), for apo and substrate-bound structures, respectively. The envelope encompasses the entire crystal structure and shows minimal fluctuations. These observations are also seen in the models when either substrate is added to the solution. Negative control variants, S61A and Q104L had similar envelopes and catalytic efficiencies to those of WT. The second-layer variants Y160F, Y160S, Y229F, Y229S, as well as the first-layer variant D231A, all had radii of gyration and envelopes similar to those of WT.

### Fluctuations predicted by MD simulations

In order to further explore the structural rearrangements observed in SAXS, particularly the large-scale conformational rearrangement in the E299Q variant suggested by SAXS measurements, we employed molecular dynamics simulations of the WT protein and 13 different variants. For this, we performed simulations with an all-atom explicit-solvent model, as well as with a simplified “structure-based” model. As described below, both sets of simulations predict large-scale fluctuations around residues 240–250 and 280–290, which is qualitatively consistent with the more extended conformation in the E299Q variant.

**Fig 7:**
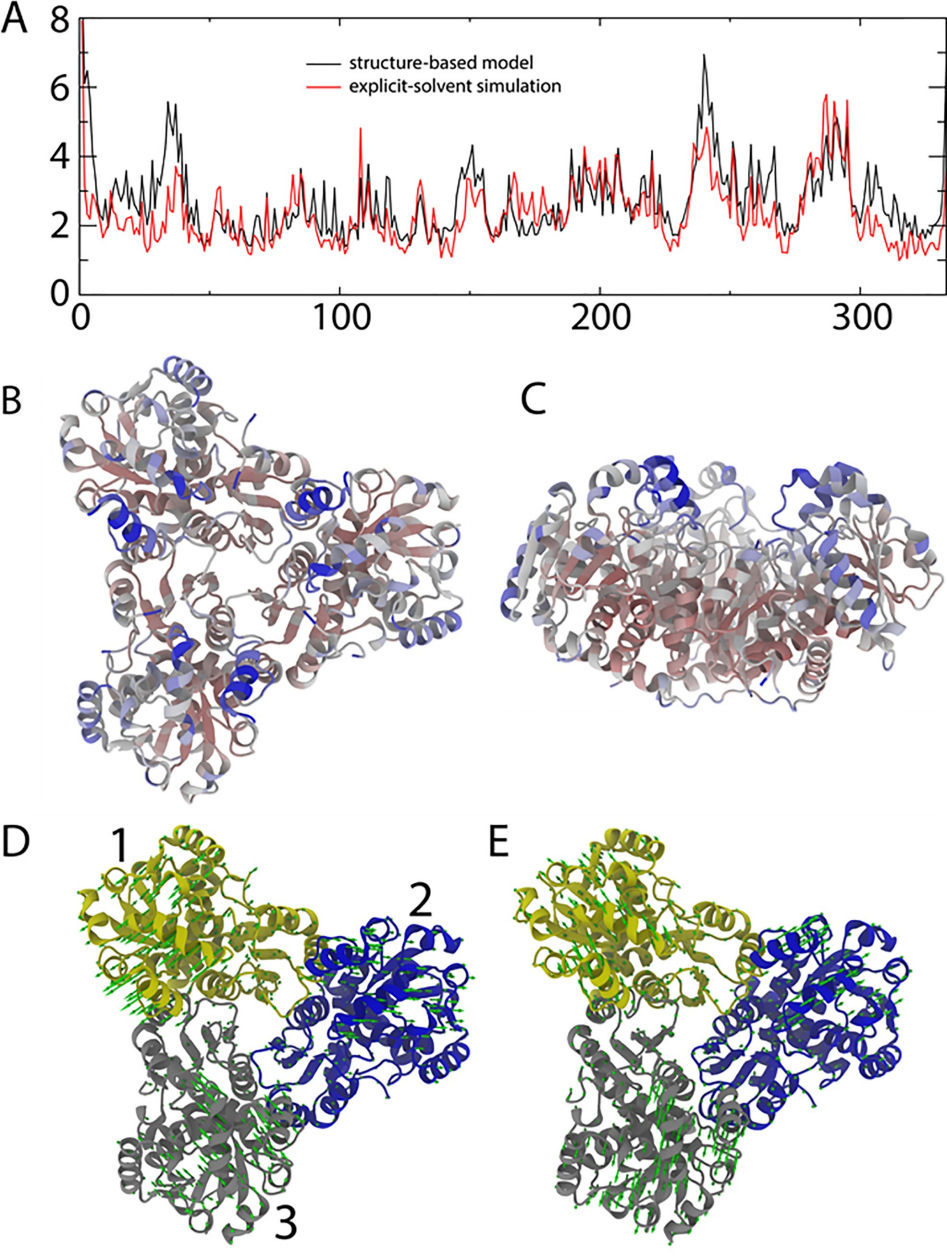
Simulations of OTC. A. Root mean-squared fluctuations (RMSF) of each residue, as obtained in simulations with an explicit-solvent model (black) and a simplified structure-based (red) model. The scale and distribution of fluctuations are similar in both models, where the largest motions are centered around residues 245 and 290. B. Top view of the OTC trimer, colored by the RMSF values obtained with the explicit-solvent model (red = 0 Å, blue = 5 Å). C. Side view OTC trimer, colored as in panel B. D. First principal component, calculated from the structure-based model. This dominant motion corresponds to an outward displacement of subunit 2, relative to subunits 1 and 3. E. The third principal component implicates simultaneous radially outward displacement of all three subunits, consistent with OTC adopting more extended structures, as implicated by SAXS measurements.

For the explicit-solvent simulations, we performed 1-μsec simulations of WT OTC and variants. Interestingly, all simulations revealed large-scale fluctuations around residues 240–250 and 280–290 (Fig 7A). However, given the large scale of the OTC trimer, the fluctuations in each subunit were not symmetric, which indicates a lack of convergence in the simulated data set. While this level of convergence precludes more rigorous comparison of fluctuations between variants with the explicit-solvent model, all subunits exhibited the largest fluctuations in the same residues. This high degree of mobility in these residues suggests that, potentially, these regions may be amenable to large-scale deformations, as indicated by SAXS. In particular, these dynamic regions may explain the appearance of more extended orientations in the SAXS envelopes (Fig 6B).

To obtain a more comprehensive description of the native-basin fluctuations, we next employed a second set of simulations using a simplified energetic model. Specifically, we applied an all-atom structure-based (Go-like) model [73] to simulate fluctuations about the native configuration. In this model, the native configuration is explicitly defined to be the potential energy minimum. The rationale for applying this representation is that the native structure represents a free-energy minimum, a necessary requirement for structure determination. Thus, every intra/inter-molecular interaction has an effective minimum that corresponds to the native conformation. This representation is similar to the harmonic approximation employed in normal mode analysis, except that the structure-based model accounts for anharmonic interactions (e.g. non-bonded interactions can form and break).

Since the all-atom structure-based model provided RMSF values that were similar to those obtained from explicit-solvent simulations (Fig 7A–7C), we next computed the spatial principal components (PC) based on the positions of the Cα atoms. Consistent with the explicit-solvent simulations, we find the first several PCs correspond to large-scale fluctuations around residues 240–250 and 280–290. In particular, PCs 1, 2 and 4 implicate outward movement of these residues in each subunit. In PC 1 (Fig 7D), domain 2 moves in opposition to domains 1 and 3. Similar extension of an individual domain is apparent in PC 2 and PC 4, consistent with the SAXS envelopes that implicate extension of the domains. In addition, PC 3 implicates concerted movement of all three domains in a radially outward direction, again similar to the SAXS observations (Fig 7E). Taken together with the explicit-solvent simulations, these results further reinforce the notion that these residues are very labile and may undergo relatively large fluctuations that may facilitate the adoption of extended domain orientations, consistent with inferences based on SAXS (Fig 6B). While this simple model is not expected to be able to capture differences due to point mutations, the high degree of conformational variability in this region suggests that such motions may be amplified, or reduced, by modest energetic changes, such as point mutations.

## Discussion

In this work, we determined the effects on enzymatic activity of mutations of POOL-predicted residues in *E*. *coli* OTC. OTC D140N and Y160S, both harboring mutations of second-layer residues, had 28-fold and 14-fold decreases in catalytic efficiencies, respectively, relative to WT, with respect to ORN. Asp140 is located just behind first-layer residue Gln136, with respect to the substrate. Asp140 is located about 8 Å from CP and about 11 Å from ORN. The carbonyl group of the amide side chain on Gln136 hydrogen bonds with the–NH_2_ group of the CP carbamate moiety and, in turn, the carboxylate side chain of Asp140 hydrogen bonds with the–NH_2_ group of the amide side chain of Gln136 (Fig 5). This hydrogen-bonding network helps to position CP for catalysis. The 28-fold decrease in catalytic efficiency for OTC D140N with respect to ORN suggests that the protonation equilibrium on the side chain of Asp140 plays some role in the catalytic process. The fluorescence difference spectrum of the D140N variant upon addition of CP (Fig 4) is similar to that of WT with CP. This indicates that the binding of CP is not impaired significantly by the mutation. The MCCE results suggest that Asp140 is electrostatically coupled to key first-layer residues Arg57 and Cys273; this is likely one of the mechanisms by which this distal residue assists catalysis. Since the protonation equilibrium of Asp140 is coupled to those of the two first-layer residues, Asp140 helps to facilitate reversible binding by Arg57 and C273, i.e. binding of substrates and release of products.

Tyr160 is located behind first-layer residue Asp231 with respect to the bound ornithine; the side chain carboxylate group of Asp231 hydrogen-bonds with the α-amino group of ornithine. The catalytic efficiency of OTC Y160F, which retains the aromatic ring but not the phenolic–OH group of Tyr, for either substrate is similar to WT. Y160S, which does not have the aromatic ring of Tyr but retains the–OH group, shows only a 1.5-fold decrease in catalytic efficiency with respect to CP, but a larger 14-fold decrease with respect to ORN. This suggests that the aromatic ring of Tyr160 makes a small contribution to catalysis, possibly simply through hydrophobic packing, providing steric bulk to position Asp231, which in turn positions ORN.

His272 is a second-layer residue, located behind first-layer residues Asp231 and Cys273 with respect to the substrates. Glu299 is a third-layer residue located behind His272. A carboxylate oxygen atom of Glu299 is 2.8 Å from a ring nitrogen atom of His 272 in the crystal structures, indicating a hydrogen-bonding or salt bridge interaction [6, 41]. The catalytic efficiency of OTC H272N with either substrate is similar to WT. However, the non-polar, non-hydrogen-bonding variant H272L shows a 120-fold decrease in catalytic efficiency with respect to ORN and a 310-fold decrease with respect to CP. The lack of an increase in T_m_ and the relatively flat fluorescence difference spectrum of OTC H272L in the presence of CP suggests decreased ability to bind CP. OTC E299Q exhibits a 51-fold decrease in catalytic efficiency with respect to ORN and a 110-fold decrease with respect to CP. The catalytic efficiency of OTC E299D for either substrate is very similar to WT; thus, retention of the charged carboxylate group retains catalytic efficiency, emphasizing the importance of the acidic side chain that interacts with His272. His272 and Glu299 are on two separate loops and likely form a hydrogen bond or salt bridge that allows for an important catalytic loop (residues 232–256) [7] to move towards the active site during catalysis. Thermal stability measurements and fluorescence difference spectra show that both E299Q and H272L have diminished CP binding. Thus, the titratable side chain of Glu299 contributes to the electrical potential at the site of reaction more than 10 Å away and His272 and Glu299 are part of a hydrogen-bonding network that facilitates CP binding and contributes to catalysis.

One of the POOL-predicted residues, Tyr229, did not show significant loss of activity upon mutation to Y229F or Y229S and no prior studies have reported evidence that it is important in catalysis. Tyr229 is a second-layer residue located behind Cys273 with respect to the substrates. We note that the side chain of Tyr229 is 3.3 Å away from the side chain of Cys273 and about 4 Å from the side chain of Tyr160. These three residues, Tyr160, Tyr229, and Cys273, are all proton donors that deprotonate to an anion with high p*K*_a_s; thus their proton transfer equilibria are coupled. They may play similar roles in the biochemistry and thus may provide some redundancy, such that the loss of the phenolic side chain of Tyr229 does not impair catalysis. Asp140, His272, and Tyr229 all interact with a C-terminal helix (residues 312–332) that is postulated to link the CP and ORN domains and play an indirect role in catalysis [77]; residues D140 and H272 have been shown here to contribute to the catalytic rate with respect to ORN.

Cys273 is a first-layer residue that when mutated to alanine was reported to result in a ~25-fold decrease in catalytic efficiency with respect to ORN [79, 81]. However, in the present study, the C273A mutation results in catalytic efficiency comparable to WT with respect to both ORN and CP. These reported differences in behavior of the C273A variant are likely attributable to different conditions, particularly differences in salt concentration or buffer conditions. While it has been demonstrated that Cys273 is important for catalytic efficiency under some conditions [79, 81], it has also been deemed to be not essential for catalysis [81], and the present results suggest that it is not a catalytic base. The strong coupling between the protonation states of Cys273 and Tyr229 will lead to expanded buffer ranges for both residues. Thus the POOL ranking for Cys273 is coincidentally elevated.

The crystal structure of OTC with a transition state mimic shows that Arg57, the positive control in this study, is involved in binding the phosphate group of CP [41] and thus is considered a first-layer residue. The relatively flat fluorescence difference spectrum upon addition of CP and the insensitivity of the T_m_ to CP for the R57A variant (Fig 4) also demonstrate the importance of R57 in CP binding. OTC R57A shows a 44-fold decrease in catalytic efficiency with respect to ORN and a 57-fold decrease with respect to CP. This is not as dramatic as the 21,000-fold reduction in turnover rate reported for R57G [2], perhaps because the introduction of glycine can result in greater conformational flexibility in a manner that impairs the ability to position CP for activity. In addition to its role in CP binding, the MCCE analysis shows that R57 is electrostatically coupled to the previously-reported active residues His133, Cys273, and Arg319, and also to Asp87, Asp140, and His272. These couplings help to expand the buffer range of Arg57, allowing its protonated and deprotonated states to be populated at neutral pH; this enables Arg57 to bind and release the phosphate group reversibly. A brief summary of the highlights of observations and conclusions about the biochemical roles of the different residues is given in Table 6.

**Table 6:** Summary of observations for variants & brief summary of biochemical roles.

Variant	Layer	CP Fold-decrease *k*_cat_/*K*_M_	ORN Fold-decrease *k*_cat_/*K*_M_	Residue roles & biochemical importance
D140N	2^nd^	5.4	28	Electrostatic coupling to R57 & C273
Y160F	2^nd^	6.7	8.2	May provide steric bulk to position D231 properly for catalysis
Y160S	2^nd^	1.5	14	
Y229F	2^nd^	3.5	2.7	Not singularly important; May be redundancy with Y160 and Cys 273
Y229S	2^nd^	1.7	1.4	
D231A	1^st^	450	580	Active 1^st^ layer residue
H272L	2^nd^	310	120	Loss of polar side chain leads to decreased ability to bind CP and decreased activity
H272N	2^nd^	4.2	3.4	
C273A	1^st^	2.8	3.2	Not significantly important under present conditions.
E299D	3^rd^	2.5	4.2	Loss of charged side chain leads to diminished CP binding; promotes conformational stability
E299Q	3^rd^	110	51	
R57A (+)	1^st^	57	44	Aids CP binding; part of an extended network of electrostatically coupled residues

We have identified four remote residues, Asp140, Tyr160, His272, Glu299, up to 9 Å away from substrates and not previously studied, to be important for OTC activity (Table 1). Spatial alignment of *E*. *coli* OTC and huOTC showed equivalent residues in huOTC as Asp175, Trp193, His302 and Glu310. The present findings suggest possible mechanisms for disease association in human variants with mutations in the aligned positions. D140N in *E*. *coli* OTC is reported here to show somewhat reduced activity, suggesting that it helps to facilitate catalysis through coupling of its protonation equilibrium to those of first-layer residues; the huOTC missense mutations D175G and D175V in the aligned position have been reported to be associated with late onset OTCD [89] and with OTCD in heterozygous females [88]. We suggest here that Y160 provides steric bulk to hold the catalytic aspartate in the optimum position; human variants W193R and W193G have been reported in heterozygous females with OTDC symptoms [91]. Evidence reported here shows that mutations at H272 lead to decreased activity and reduced CP binding; mutations H302Y and H302R in the aligned human residue are associated with neonatal OTCD [85, 89]. Present results show apparent decreased ability to bind CP in the third-shell variant E299Q and significantly increased radius of gyration in the SAXS data, suggesting partial unfolding; the E310G variant in the aligned position in huOTC has been reported to be associated with late onset OTCD [91].

The mutations H272L and E299Q likely perturb binding of CP. The solution scattering results on the OTC variants D140N, H272L, H272N, C273A, E299D, and E299Q and on R57A (+) suggest that these residues play a key role in conformation and dynamics, as these variants show partial unfolding or partial separation of domains in solution. PCA analysis from the MD simulations provides further evidence of these conformational shifts. This work establishes the importance of certain second-layer residues in the facilitation of substrate binding and catalysis. It also suggests the importance of investigating equivalent residues in huOTC to understand how these residues may play a role in OTC deficiency.

## Abbreviations

OTC: ornithine transcarbamoylase
CIT: citrulline
P_i_: inorganic phosphate
CP: carbamoyl phosphate
ORN: ornithine
PALO: N-phosphonoacetyl-L-ornithine
huOTC: human ornithine transcarbamoylase
LPC: Ligand-Protein Contacts
CSU: Contacts of Structrual Units
TSC: thiosemicarbazide
MCCE: MultiConformer Continuum Electrostatics
